# ORF3a Protein of Severe Acute Respiratory Syndrome Coronavirus 2 Inhibits Interferon-Activated Janus Kinase/Signal Transducer and Activator of Transcription Signaling *via* Elevating Suppressor of Cytokine Signaling 1

**DOI:** 10.3389/fmicb.2021.752597

**Published:** 2021-09-28

**Authors:** Rong Wang, Xiaofeng Yang, Mingke Chang, Ziyang Xue, Weirong Wang, Liang Bai, Sihai Zhao, Enqi Liu

**Affiliations:** Laboratory Animal Center, Xi’an Jiaotong University Health Science Center, Xi’an, China

**Keywords:** SARS-CoV-2, JAK/STAT signaling, accessory protein ORF3a, SOCS1, Janus kinase 2 (JAK2), ubiquitin-proteasomal degradation

## Abstract

Coronavirus disease 2019 (COVID-19) has caused a crisis to global public health since its outbreak at the end of 2019. Severe acute respiratory syndrome coronavirus 2 (SARS-CoV-2), the pathogen of COVID-19, appears to efficiently evade the host immune responses, including interferon (IFN) signaling. Several SARS-CoV-2 viral proteins are believed to involve in the inhibition of IFN signaling. In this study, we discovered that ORF3a, an accessory protein of SARS-CoV-2, inhibited IFN-activated Janus kinase (JAK)/signal transducer and activator of transcription (STAT) signaling *via* upregulating suppressor of cytokine signaling 1 (SOCS1), a negative regulator of cytokine signaling. ORF3a induced SOCS1 elevation in a dose- and time-dependent manner. RNAi-mediated silencing of SOCS1 efficiently abolished ORF3a-induced blockage of JAK/STAT signaling. Interestingly, we found that ORF3a also promoted the ubiquitin-proteasomal degradation of Janus kinase 2 (JAK2), an important kinase in IFN signaling. Silencing of SOCS1 by siRNA distinctly blocked ORF3a-induced JAK2 ubiquitination and degradation. These results demonstrate that ORF3a dampens IFN signaling *via* upregulating SOCS1, which suppressed STAT1 phosphorylation and accelerated JAK2 ubiquitin-proteasomal degradation. Furthermore, analysis of ORF3a deletion constructs showed that the middle domain of ORF3a (amino acids 70–130) was responsible for SOCS1 upregulation. These findings contribute to our understanding of the mechanism of SARS-CoV-2 antagonizing host antiviral response.

## Introduction

The pandemic of coronavirus disease 2019 (COVID-19) has caused a crisis to global public health. Mild to severe respiratory illness is the typical clinical symptom of COVID-19. Hypercytokinemia and acute respiratory distress syndrome may eventually lead to a long-term reduction in lung function and death (Ramasamy and Subbian, 2021). The causative agent of the disease is severe acute respiratory syndrome coronavirus 2 (SARS-CoV-2), an enveloped, positive-sense, single-stranded RNA virus belonging to the genus *Betacoronaviruses* in the family of *Coronaviridae*. The genome of SARS-CoV-2 is approximately 30 kb in length and encodes 4 structural proteins [spike (S), envelope (E), membrane (M), and nucleocapsid (N) proteins], 16 non-structural proteins (nsp1–16), and 7 accessory proteins (ORF3a, 3b, 6, 7a, 7b, 8, and 10) (Kim et al., 2020). SARS-CoV-2 appears to be highly transmissible in the human population, which might be associated with an effective evasion of the host’s innate immunity (Amor et al., 2020).

Interferons (IFNs) are important cytokines of the host immune system and are mainly classified into three types: I (IFNα, IFNβ), II (IFNγ), and III (IFNλ). Once the virus initiates infection, host pattern-recognition receptors will detect viral nucleic acids and induce the production of IFNs (Gonzalez-Navajas et al., 2012). SARS-CoV-2 not only suppress IFNs induction (Blanco-Melo et al., 2020; Chen et al., 2020; Hadjadj et al., 2020; Hu et al., 2020) but also impair IFN-mediated antiviral response *via* dampening the activation of Janus kinase (JAK)/signal transducer and activator of transcription (STAT) signaling (Hayn et al., 2021). In the canonical pathway of IFN-activated JAK/STAT signaling, IFNs bind to their receptors on the cell surface to activate JAKs, which then recruit and phosphorylate STATs. The phosphorylated STATs form homo- or heterodimers, followed by translocation into the nucleus and bind with the corresponding responses elements, such as interferon-stimulated response element (ISRE), to trigger the transcription of IFN-stimulated genes (ISGs), which result in the establishment of an antiviral state (Darnell et al., 1994; Stark et al., 1998).

It is reported that the non-structural proteins and accessory proteins of SARS-CoV-2 are involved in modulating the JAK/STAT signaling to facilitate infection and pathogenesis (Lei et al., 2020; Miorin et al., 2020; Xia et al., 2020; Yuen et al., 2020; Hayn et al., 2021; Kumar et al., 2021; Yadav et al., 2021). However, the effects of accessory proteins, such as ORF3a, on IFNα/β-induced ISRE-promoter activation are inconsistent (Lei et al., 2020; Xia et al., 2020; Kumar et al., 2021). The objective of this study was to determine the role of ORF3a on IFN-activated signaling and then elucidate the underlying mechanism. By utilizing three different detection methods, we found that SARS-CoV-2 ORF3a had a significant inhibitory effect on IFN-activated JAK/STAT signaling. ORF3a suppressed the phosphorylation and nuclear translocation of STAT1 *via* elevating suppressor of cytokine signaling 1 (SOCS1), a negative feedback regulator of JAK/STAT signaling (Yoshimura et al., 2007). ORF3a also induced Janus kinase 2 (JAK2) ubiquitin-proteasomal degradation by SOCS1. The middle domain of ORF3a [amino acids (aa) 70–130] is crucial for it to function. This discovery provides further insight into SARS-CoV-2 interference with IFN-activated signaling.

## Materials and Methods

### Cells and Chemicals

HEK293T cells (ATCC) were maintained in Dulbecco’s Modified Eagle Medium (DMEM) supplemented with 10% fetal bovine serum (FBS).

For interferon stimulation, recombinant human IFNα-2a (Cat. No. Z03003-50, GenScript) was added to the cultured cells at a final concentration of 500 U/mL (unless stated differently in section “Results” and the figure legends). The cells were harvested at time points indicated in the results or the figure legends for further analysis.

MG132 (Cat. No. S1748, Beyotime Biotechnology), a proteasome inhibitor, was added to cells at 10 μM final concentration for 6 h before the cells being harvested for further analysis. Cycloheximide (CHX) (Cat. No. SC0353, Beyotime Biotechnology) was added to cultured cells at a final concentration of 100 μg/mL to block protein translation to determine the half-life of JAK2. The cells were harvested at the time points indicated in section “Results” or the figure legends for immunoblotting.

Lipofectamine^TM^ 2000 Transfection Reagent (Cat. No. 11668-027, Invitrogen) and HiPerFect Transfection Reagent (Cat. No. 301704, QIAGEN) were used to transfect plasmid DNA and siRNA, respectively, into the cells according to the manufacturers’ instructions.

### Plasmids and siRNA

Severe acute respiratory syndrome coronavirus 2 ORF3a sequence was synthesized by GenScript according to the sequence of the strain Wuhan-Hu-1 (GenBank accession NC_045512.2). Then, it was subcloned to pCAGEN-Flag vector as described (Wang et al., 2013a; Yang et al., 2017). The resulting recombinant plamsid was confirmed by restriction enzyme digestion and DNA sequencing. Its overexpression in transfected cells was confirmed by indirect immunofluorescence microscopy and immunoblotting.

Sequences of siRNA targeting human SOCS1 (si-SOCS1) and non-targeting control siRNA (NC) are 5′-CACGCACUUCCGCACAUUCTT-3′ (Hong et al., 2009) and 5′-UUCUCCGAACGUGUCACGUTT-3′, respectively. The siRNA duplexes were synthesized and purified by Sangon Biotech (Shanghai, China).

### Luciferase Reporter Assay

The assay was conducted and analyzed as described previously (Wang et al., 2013a). Briefly, HEK293T cells were transfected with the ISRE-firefly luciferase reporter plasmid and testing plasmids. Renilla luciferase vector pGL4.74 hRL-TK was also included for normalization. Empty vector (EV) of testing plasmids was included as a control. IFNα was added to the cells at 500 U/mL the next day. At 20 h after the IFN treatment, the cells were detected by Dual-Glo luciferase assay system (Cat. No. E2920, Promega) following the manufacturer’s instructions. The ISRE reporter activity was normalized against Renilla reporter values. Relative folds of luciferase activity in comparison with mock-treated control were shown.

### Immunofluorescence Assay

Cells were seeded into wells of a culture plate with coverslips, incubated overnight, and then treated as the descriptions indicated in the figure legends. Immunofluorescence assay (IFA) with antibodies against STAT1 [Cat. No. 14994T, Cell Signaling Technology (CST)] and Flag (Cat. No. 66008, Proteintech) was done as described (Wang et al., 2013a). The specific reactions were detected by the conjugated secondary antibodies: goat anti-mouse IgG (H&L) Cy3 (Cat. No. ab97035, Abcam) and anti-rabbit IgG (H&L), F(ab′)2 Fragment (Alexa Fluor^®^ 488 Conjugate) (Cat. No. 4412S, CST). The coverslips were mounted onto slides using DAPI Fluoromount-G (Cat. No. 0100, Southern Biotech). The fluorescence signal was observed under confocal fluorescence microscopy (Nikon C2), and images were taken with NIS-Elements version 4.0 (Nikon).

### RNA Isolation and Real-Time PCR

According to the manufacturer’s instructions, total RNA was isolated from cells with RNAiso Plus (Cat. No. 9108, Takara). Reverse transcription and real-time quantitative PCR (RT-qPCR) were conducted as previously described (Wang et al., 2013b). Transcript of ribosomal protein L32 (RPL32) was also amplified and used to normalize the total amount of input RNA. Primers used in this study were listed in Table 1. Relative transcript levels were quantified by the 2^–ΔΔCT^ threshold cycle method (Livak and Schmittgen, 2001) and were shown as fold changes relative to a control.

**TABLE 1:** List of primers for real-time PCR.

**Primer^*a*^**	**Sequences (5′-3′)**	**Target gene**
RPL32-F	ACAAAGCACATGCTGCCCAGTG	RPL32
RPL32-R	TTCCACGATGGCTTTGCGGTTC	RPL32
ISG15-F	CACCGTGTTCATGAATCTGC	ISG15
ISG15-R	CTTTATTTCCGGCCCTTGAT	ISG15
ISG56-F	CCTCCTTGGGTTCGTCTACA	ISG56
ISG56-R	GGCTGATATCTGGGTGCCTA	ISG56
JAK2-F	CCAGATGGAAACTGTTCGCTCAG	JAK2
JAK2-R	GAGGTTGGTACATCAGAAACACC	JAK2
SOCS1-F	TTCGCCCTTAGCGTGAAGATGG	SOCS1
SOCS1-R	TAGTGCTCCAGCAGCTCGAAGA	SOCS1
SHP1-F	TTGACCACAGCCGAGTGATCCT	SHP1
SHP1-R	CTGGCGATGTAGGTCTTAGCGT	SHP1
SHP2-F	GACTTTTGGCGGATGGTGTTCC	SHP2
SHP2-R	CGGCGCTTTCTTTGACGTTCCT	SHP2

*^a^F, a forward primer; R, a reverse primer.*

### Immunoblotting

Proteins samples were separated by sodium dodecyl sulfate-polyacrylamide gel electrophoresis (SDS-PAGE) and analyzed by immunoblotting as described previously (Wang et al., 2013b). The separated proteins were transferred onto PVDF membrane and probed with antibodies against STAT2 (Cat. No. ab32367, Abcam), phosphorylated STAT1 at tyrosine-701 (STAT1-Y701) (Cat. No. ab109457, Abcam), STAT1 (Cat. No. 14994T, CST), JAK1 (Cat. No. ab133666, Abcam), JAK2 (Cat. No. ab108596, Abcam), TYK2 (Cat. No. A2128, ABclonal), SOCS1 (Cat. No. AF8022, Beyotime Biotechnology), β-actin (Cat. No. ab8227, Abcam), α-tubulin (Cat. No. ab15246, Abcam), and Flag. The antibodies bound on the membrane were detected with secondary antibodies conjugated with horseradish peroxidase (Cat. No. 31460, Thermo Fisher Scientific), followed by revealing with a chemiluminescence substrate. The luminescence signal was recorded digitally by using a Chemi-Doc XRS imaging system (Bio-Rad). Digital image acquisition and analysis were conducted using the Quantity One program (Bio-Rad).

### Immunoprecipitation

Immunoprecipitation (IP) was conducted as previously described (Wang et al., 2013b). Cells were lysed with a lysis buffer (50 mM Tris, pH 7.4, 150 mM NaCl, 0.2 mM EDTA, 2 mM EGTA, 0.5% Igepal CA-630, 10% glycerol, 1 mM sodium vanadate) supplemented with 2.53 μM ubiquitin aldehyde, a specific inhibitor of ubiquitin C-terminal hydrolases (Cat. No. U-201-050, Boston Biochem), and complete EDTA-free, a protease inhibitor (Cat. No. 4693132001, Roche). The lysate was clarified by centrifugation at 14,000 × *g* for 5 min at 4°C. The supernatant was transferred to a new tube and incubated with JAK2 antibody, then with dynabeads protein G (Cat. No. 10003D, Invitrogen). The final pellet from IP was subjected to elution with 100 mM glycine solution, pH 3.0. The IP samples were subjected to immunoblotting with antibodies against ubiquitin (Ub) (Cat. No. SC-8017, Santa Cruz Biotechnology), SOCS1, and JAK2.

### Statistical Analysis

Data are expressed as mean ± SE. Student’s *t*-tests (for comparison between treatment group and control group) or one-way analysis of variance (ANOVA) (for comparisons of ≥3 groups) followed by Tukey *post hoc* tests were used for the statistical analyses. GraphPad Prism 5 software was used. A two-tailed *P*-value of less than 0.05 was considered significant.

## Results

### Severe Acute Respiratory Syndrome Coronavirus 2 Accessory Protein ORF3a Inhibits Interferon-Activated Janus Kinase/Signal Transducer and Activator of Transcription Signaling

**FIGURE 1:**
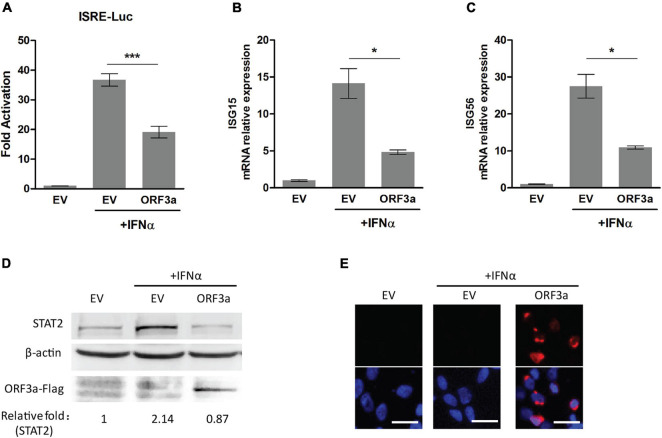
Severe acute respiratory syndrome coronavirus 2 accessory protein ORF3a inhibits IFN-activated JAK/STAT signaling. **(A)** ISRE luciferase reporter assay. HEK293T cells were cotransfected with ISRE-Luc reporter plasmid, pGL4.74 hRL-TK plasmid, and empty vector (EV) or expression plasmid of SARS-CoV-2 ORF3a. At 24 h post-transfection, the cells were mock-treated or treated with IFNα (500 U/mL) for 20 h and then subjected to Dual-Luciferase reporter assay. The fold activation was determined compared to that of the EV with mock-treated cells. **(B–D)** The expression levels of ISG15, ISG56, and STAT2 in HEK293T cells with ORF3a plasmid transfection. HEK293T cells were transfected with a plasmid expressing ORF3a for 24 h. Then, the cells were treated with IFNα. ISG15 **(B)** and ISG56 **(C)** mRNA levels were determined at 13 h after the IFN treatment by RT-PCR. STAT2 levels were detected at 20 h after the IFN treatment by immunoblotting **(D)**. Relative levels of STAT2 are shown as folds below the images after normalization with β-actin in densitometry analysis. **(E)** Expression of ORF3a plasmid in HEK293T cells was confirmed by immunofluorescence staining with antibody against Flag. The upper panel shows ORF3a expression, and the lower panel shows an overlay of protein expression and DAPI staining. Bars in the images denote 20 μm. Significant differences from the IFN-treated EV control are denoted by “*” and “***”, which indicate *P* < 0.05 and *P* < 0.001, respectively. Error bars represent standard errors of the results of repeated experiments.

Severe acute respiratory syndrome coronavirus 2 encodes multiple accessory proteins, some of which were speculated to antagonize JAK/STAT signaling. In this study, for determining the effect of accessory protein ORF3a on IFN signaling, an ISRE luciferase reporter assay was conducted. Compared to mock-treated control, the cells transfected with EV or ORF3a plasmid had the IFN-induced ISRE reporter expression levels at 36.71 and 19.12-fold, respectively (Figure 1A). ORF3a significantly reduced ISRE reporter expression compared to the IFN-treated EV control.

In addition, since type I IFN signaling leads to elevated expression of a myriad of proteins encoded by IFN-responsive genes, such as ISG15, ISG56, and STAT2 (Darnell et al., 1994; Stark et al., 1998), we also detected the expression levels of ISG15, ISG56, and STAT2 by RT-qPCR or immunoblotting. Compared to mock-treated control, IFN treatment led to the transcript level of ISG15 increase 4.84-fold in ORF3a transfected cells, which was significantly lower than the 14.14-fold increase in IFN-treated EV-transfected cells (Figure 1B). Similarly, the ISG56 transcript levels after IFN treatment were upregulated to 10.91-fold in ORF3a transfected cells, which was considerably lower than the 27.5-fold increase in IFN treated cells with EV transfection (Figure 1C). Besides, compared with the IFN-treated cells with EV, the cells expressing ORF3a had considerably lower levels of STAT2 (Figure 1D). The expression of the ORF3a plasmid in HEK293T cells was confirmed by immunoblotting and IFA (Figures 1D,E). The results above demonstrated that ORF3a had a significant inhibitory effect on IFN-activated JAK/STAT signaling.

### ORF3a Dampens Interferon-Activated Signaling *via* Restraining STAT1 Phosphorylation and Nuclear Translocation

**FIGURE 2:**
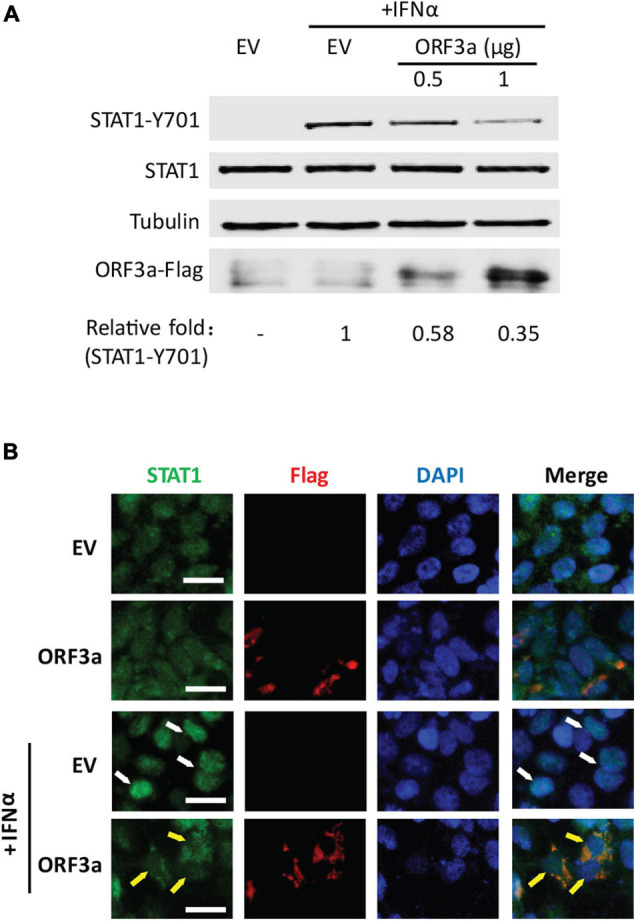
ORF3a dampens IFN-activated signaling *via* restraining STAT1 phosphorylation and nuclear translocation. **(A)** ORF3a inhibits IFN-induced phosphorylation of STAT1 in HEK293T cells. Cells were transfected with 0.5 or 1 μg of ORF3a plasmid. Forty-eight hours later, the cells were stimulated with IFNα at 500 U/mL for 0.5 h, then harvested for immunoblotting with antibodies against STAT1-Y701, STAT1, tubulin, and Flag. Relative levels of STAT1-Y701 are shown as folds below the images after normalization with tubulin. **(B)** ORF3a restrains nuclear translocation of STAT1. The transfected cells were treated with IFNα for 0.5 h and then fixed with paraformaldehyde for immunofluorescence assay with antibodies against STAT1 (green) and Flag (red). DAPI staining of nuclear DNA is also shown. Arrows were added to show the STAT1 in the nucleus (white) or cytoplasm (yellow). Bars in images denote 20 μm.

STAT1 is a crucial player in the JAK/STAT signaling. Once type I IFNs bind to their receptors, STAT1 phosphorylation, and nuclear translocation will be quickly initiated. To investigate whether SARS-CoV-2 ORF3a impairs IFN-activated signaling *via* inhibiting IFN-induced STAT1 activation, we tested the phosphorylation and nuclear translocation of STAT1 in HEK293T cells 0.5 h after IFNα treatment. In mock-treated cells, the phosphorylation of STAT1 at tyrosine 701 (STAT1-Y701) was below detection level. After IFNα treatment, STAT1-Y701 level was greatly increased in cells transfected with EV, while ORF3a expression considerably reduced the phosphorylation level of STAT1 (Figure 2A). Moreover, the inhibitory effect of ORF3a on STAT1 phosphorylation is in a dose-dependent manner. Along with the increase of ORF3a expression, STAT1-Y701 level further decreased (Figure 2A). However, ORF3a had minimal effect on the total STAT1 levels (Figure 2A).

The IFN-induced STAT1 nuclear translocation was also observed in the presence or absence of ORF3a. In the cells with ectopic ORF3a expression, most STAT1 remained in the cytoplasm, whereas in the cells transfected with an EV, most STAT1 were translocated into the nucleus (Figure 2B). The results above indicated that ORF3a interfered with the JAK/STAT pathway by inhibiting STAT1 phosphorylation and nuclear translocation.

### ORF3a Upregulates SOCS1, Which Inhibits Interferon-Activated Signaling Transduction

**FIGURE 3:**
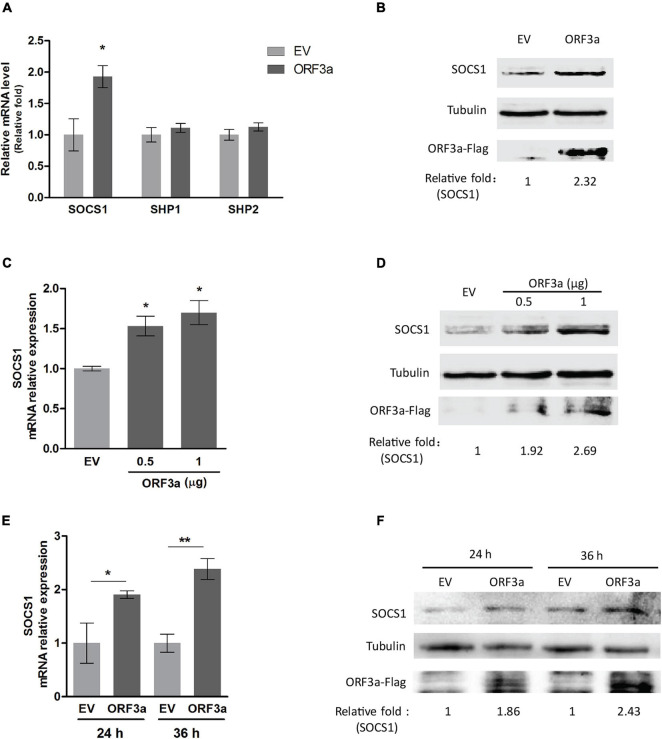
ORF3a increases SOCS1 expression. **(A)** ORF3a increases the transcription level of SOCS1 instead of SHP1 and SHP2. HEK293T cells were transfected with empty vector (EV) or ORF3a plasmid. At 24 h after transfection, the cells were harvested for RT-qPCR. **(B)** ORF3a increases the protein level of SOCS1. Cells were treated similarly as A, then harvested at 36 h after transfection for immunoblotting. **(C,D)** ORF3a induces SOCS1 upregulation in a dose-dependent manner. HEK293T cells were transfected with incremental amounts of ORF3a plasmid for determining SOCS1 at mRNA **(C)** and protein **(D)** levels. **(E,F)** ORF3a increases SOCS1 expression along with time extension. The cells transfected with EV or ORF3a plasmid were harvested at 24 and 36 h after transfection. The transcripts **(E)** and proteins **(F)** of SOCS1 were detected. Error bars represent standard errors of the results of repeated experiments. Significant differences from the EV-transfected control cells are denoted by “*” and “**”, which indicates *P* < 0.05 and *P* < 0.01, respectively. In densitometry analysis, relative levels of SOCS1 in comparisons with EV samples are shown as folds below the images after normalization with tubulin.

The JAK/STAT signaling is regulated by various negative feedback regulators, including SOCS proteins, protein inhibitors of activated STAT (PIAS) proteins, and protein tyrosine phosphatases (PTPs) (Shuai and Liu, 2003). PIAS proteins act by blocking STAT-DNA binding or STAT-mediated transactivation, while SOCS and PTPs (such as SHP1, SHP2) mainly function by modulating the phosphorylation of JAKs or STATs (Shuai and Liu, 2003). Because ORF3a dampened the IFNα-induced phosphorylation of STAT1, we focused on the main negative regulators related to STAT1 phosphorylation, such as SOCS1, SHP1, and SHP2. Our results showed that ORF3a significantly increased the expression of SOCS1 but had minimal effect on the expression of SHP1 or SHP2 (Figures 3A,B). Moreover, ORF3a considerably increased the transcript and protein levels of SOCS1 in a dose- and time-dependent manner (Figures 3C–F). Therefore, we speculated that the ORF3a-induced JAK/STAT signaling blockage could be due to the elevated SOCS1.

**FIGURE 4:**
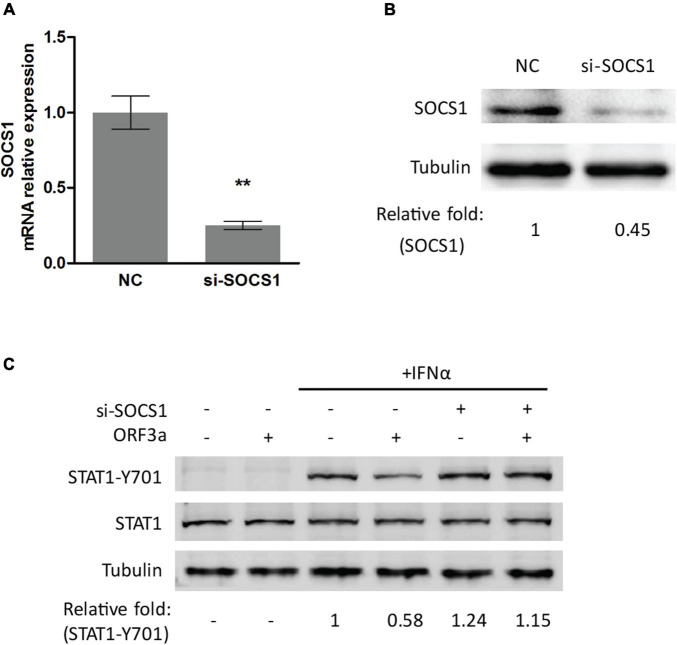
Silencing of SOCS1 abolishes the inhibitory effect of ORF3a on IFN signaling transduction. **(A,B)** SOCS1 mRNA and protein levels in cells transfected with siRNA control (NC) or siRNA against SOCS1 (si-SOCS1). HEK293T cells were transfected with NC or si-SOCS1 (100 nM). *Forty-eight hours* later, the cells were harvested for RT-qPCR **(A)** and immunoblotting **(B)**. Significant differences from the NC-transfected control cells are denoted by “**”, which indicates *P* < 0.01. **(C)** Silencing of SOCS1 restored the STAT1 activation in cells transfected with ORF3a. Cells were cotransfected with empty vector or ORF3a plasmid along with NC or si-SOCS1. At 48 h after transfection, the cells were stimulated with IFNα for 0.5 h and then harvested for immunoblotting with antibodies against STAT1-Y701, STAT1, and tubulin. Relative levels of proteins are shown as folds below the images after normalization with tubulin.

To test the speculation, we conducted RNAi-mediated silencing of SOCS1 with siRNA. The knockdown effect of si-SOCS1 on SOCS1 expression in transcription and protein levels was confirmed by RT-qPCR and immunoblotting, respectively. It showed that si-SOCS1 efficiently reduced SOCS1 RNA and protein levels (Figures 4A,B). Next, we determined the phosphorylation level of STAT1 in cells transfected with ORF3a plasmid and si-SOCS1. The cells treated with non-targeting siRNA (NC) were used as a control. It showed that siRNA silencing of SOCS1 distinctly abolished the inhibitory effect of ORF3a on JAK/STAT signaling activation. In response to the IFNα stimulation, compared to the ORF3a transfected cells without SOCS1 silencing, the cells with ORF3a and si-SOCS1 co-transfection had a considerably higher level of STAT1-Y701, which was similar to the cells transfected with EV and NC (Figure 4C). The result above demonstrates that ORF3a upregulates SOCS1, which inhibits IFN-activated signaling transduction.

### ORF3a Reduces Janus Kinase 2 *via* Ubiquitin-Proteasomal Degradation

**FIGURE 5:**
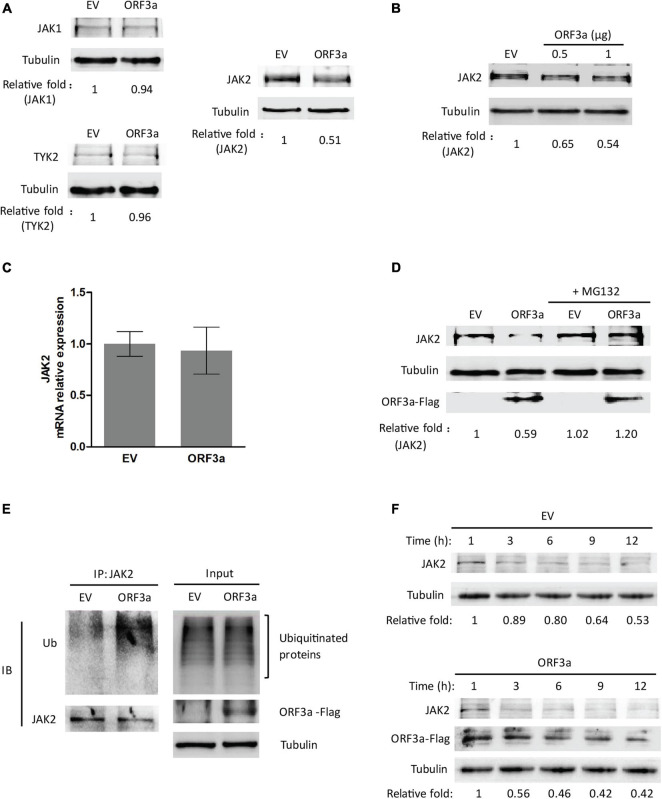
ORF3a reduces JAK2 expression *via* the proteasome pathway. **(A)** ORF3a reduces the JAK2 level, but not JAK1 and TYK2. HEK293T cells were transfected with empty vector (EV) or ORF3a plasmid, then harvested for immunoblotting at 36 h post-transfection. **(B)** ORF3a decreases JAK2 in a dose-dependent manner. Cells were transfected with incremental amounts of ORF3a plasmid and then harvested for immunoblotting to determine the JAK2 level. **(C)** JAK2 mRNA level is not affected by ORF3a expression. HEK293T cells were transfected with EV or ORF3a plasmid. At 24 h after transfection, the cells were harvested for RNA extraction and RT-qPCR. Error bars represent standard errors of the results of repeated experiments. **(D)** JAK2 reduction in ORF3a-expressed cells is restored by MG132 treatment. HEK293T cells were transfected with EV or ORF3a plasmid. At 30 h after transfection, the cells were treated with MG132 for 6 h and then harvested for immunoblotting with antibodies against JAK2, Flag, and tubulin. Relative levels of JAK2 are shown as folds below the images after normalization with tubulin in densitometry analysis. **(E)** ORF3a increases JAK2 ubiquitination. The EV or ORF3a transfected cells were lysed with IP lysis buffer, followed by IP with JAK2 antibody. The input and IP products were subjected to immunoblotting with antibodies against ubiquitin (Ub), JAK2, Flag, and tubulin. **(F)** JAK2 half-life is shortened in the presence of ORF3a. HEK293T cells transfected with ORF3a plasmid were treated with cycloheximide (CHX) and harvested at indicated times (h) for immunoblotting. EV transfected cells were included as a control. Relative levels of JAK2 are shown as folds below the images after normalization with tubulin.

Janus kinases, including JAK1, JAK2, and TYK2, are responsible for STATs phosphorylation (Yamaoka et al., 2004). Since SARS-CoV-2 ORF3a suppressed STAT1 phosphorylation, we wondered whether ORF3a interferes with JAKs. Therefore, the effects of ORF3a on JAKs expressions were determined. Compared with the EV-transfected control, the JAK2 level in the cells with ORF3a transfection considerably declined, while JAK1 and TYK2 were minimally changed (Figure 5A). Moreover, the JAK2 was decreased by ORF3a in a dose-dependent manner (Figure 5B). It indicated that ORF3a reduced JAK2 expression, which may dampen JAK2 activation.

We speculated that the JAK2 reduction could be due to decreased transcription and/or translation and accelerated protein degradation. RT-qPCR was used to determine the transcript levels of JAK2 in the cells with ORF3a expression. The mRNA levels of JAK2 in cells with and without ORF3a transfection were similar, indicating that the reduction of JAK2 expression was not due to its mRNA level (Figure 5C). Then, we determined whether the reduction of JAK2 was due to degradation by the ubiquitin-proteasomal pathway. MG132, a proteasome inhibitor, was added to the cells 30 h after ORF3a transfection. Six hours later, the cells were harvested for immunoblotting. MG132 treatment of cells with ORF3a expression restored JAK2 to a level similar to that in cells with the EV control (Figure 5D). These results indicated that the reduction of JAK2 in the ORF3a-expressed cells was due to degradation by the ubiquitin-proteasomal pathway.

### ORF3a Increases Janus Kinase 2 Ubiquitination and Shortens Its Half-Life

Since MG132 treatment restored JAK2 levels, we predicted that the JAK2 ubiquitination levels in the cells with ORF3a expression would increase. HEK293T cells were transfected with an EV or ORF3a plasmid. Thirty-six hours later, the cells were lysed for IP with an antibody against JAK2. Immunoblotting with ubiquitin antibody was then performed to detect ubiquitinated JAK2. The results showed that ubiquitinated JAK2 in the cells with ORF3a appeared as a smear, as expected, and that its density was stronger than in the cells transfected with EV (Figure 5E), indicating that there was more ubiquitinated JAK2 in the cells with ORF3a expression. In addition, input blotting showed that the total ubiquitination levels in the cells with ORF3a expression were similar to those in control cells (Figure 5E). It indicated that ORF3a did not affect total ubiquitination levels in the cells.

As ORF3a increased JAK2 ubiquitination, we speculated that the half-life of JAK2 would be shortened. Therefore, the half-life of JAK2 in cells with or without ORF3a expression was determined. HEK293T cells were transfected with an EV or ORF3a plasmid. Twenty-four hours later, the cells were treated with a translation inhibitor, CHX, at a final concentration of 100 μg/mL. The cells were then harvested at different time points for immunoblotting. In the presence of ORF3a, the JAK2 levels decreased at a higher rate than the cells with the EV (Figure 5F). The JAK2 level in the cells with ORF3a expression at 3 h after the CHX treatment was reduced to 0.56-fold, while it remained at 0.89-fold in cells with the EV (Figure 5F). In the control cells, the JAK2 half-life was about 12 h post CHX treatment. The result indicated that ORF3a shortened the half-life of JAK2 from approximately 12 h to 3 h, which is consistent with our data showing increased ubiquitination of JAK2 in the cells with ORF3a expression.

### SOCS1 Mediates ORF3a-Induced Janus Kinase 2 Ubiquitination

It is known that SOCS1 is characterized by a SOCS-box at its C-terminal end, which functions to recruit the E3 ubiquitin ligase adaptors elongins-B/C, and thereby catalyzes the ubiquitination of target proteins (Liau et al., 2018). This study found that ORF3a upregulated SOCS1 expression. Therefore, we speculated that the ORF3a-induced JAK2 reduction could be due to the elevated SOCS1.

**FIGURE 6:**
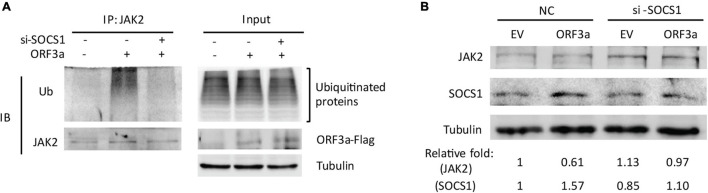
Silencing of SOCS1 diminishes ORF3a-induced JAK2 degradation. **(A)** Knockdown of SOCS1 lessens the elevation of JAK2 ubiquitination induced by ORF3a, while ubiquitination levels of total proteins in input were similar. Cells were cotransfected with empty vector (EV) or ORF3a plasmid along with NC or si-SOCS1. Thirty-six hours later, the cells were harvested the whole cell lysate (input) with IP lysis buffer. IP was conducted with JAK2 antibody. The input and IP products were detected by immunoblotting with Ub, JAK2, Flag, and tubulin antibodies. **(B)** Silencing of SOCS1 reverses ORF3a-induced JAK2 reduction. Cells were cotransfected with EV or ORF3a and NC or si-SOCS1, and at 36 h after transfection, harvested for immunoblotting. Relative levels of proteins are shown as folds below the images after normalization with tubulin.

To test the speculation, we determined the ubiquitination level of JAK2 in cells transfected with ORF3a plasmid and si-SOCS1. The cells treated with non-targeting siRNA (NC) were used as a control. Compared to the ORF3a-transfected cells with NC treatment, siRNA silencing of SOCS1 distinctly weakened JAK2 ubiquitination levels (Figure 6A). The JAK2 ubiquitination levels in ORF3a and si-SOCS1 cotransfected cells were similar to those in the cells cotransfected with EV and NC (Figure 6A). Immunoblotting of input showed a minimal change of total ubiquitination levels in the cells with NC or si-SOCS1 treatment (Figure 6A). It demonstrates that SOCS1 mediates ORF3a-induced JAK2 ubiquitination.

In addition, the expression levels of JAK2 in cells with si-SOCS1 treatment were also detected. SOCS1 silencing abolished the ORF3a-mediated JAK2 reduction, shown by the JAK2 levels similar to NC-treated EV control (Figure 6B). The results above indicate that ORF3a upregulated SOCS1 expression, which then accelerated JAK2 ubiquitin-proteasomal degradation.

### The Middle Domain of ORF3a (aa 70–130) Appears to Be Responsible for the SOCS1 Elevation

**FIGURE 7:**
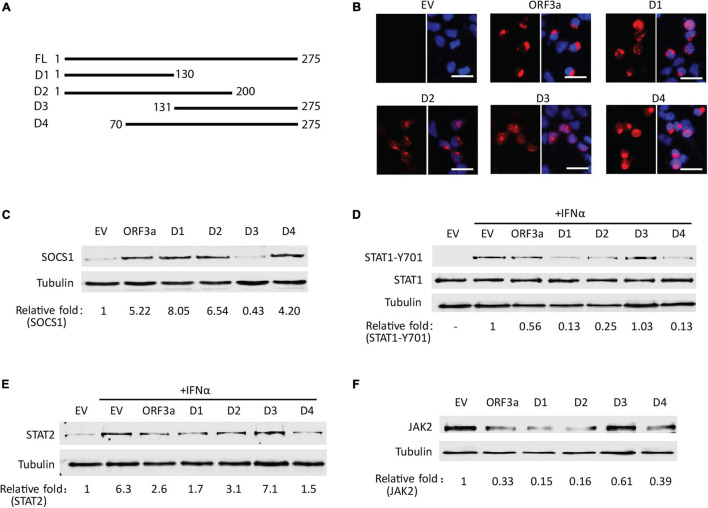
The middle domain of ORF3a appears to be associated with the JAK2 reduction and IFN signaling inhibition. **(A)** Schematic illustration of full-length (FL) and truncated constructs (D1–D4) of ORF3a. The names of the fragments are indicated on the left. The numbers above the lines indicate starting and ending amino acid positions of ORF3a for the constructs. **(B)** Immunofluorescence assay showing expression of ORF3a deletion constructs in HEK293T cells. In each panel, the left image shows the expression of EV, ORF3a, or the deletion construct, and the right image shows the overlay with DAPI staining. **(C)** The levels of SOCS1 in HEK293T cells with expression of ORF3a deletion constructs. The cells were transfected with full-length ORF3a and truncation constructs. At 48 h after transfection, cells were harvested for immunoblotting with antibodies against SOCS1 and tubulin. **(D,E)** The effect of ORF3a deletion constructs on IFN signaling activation. At 36 h after transfection, the cells were treated with IFNα for another 0.5 or 20 h, then harvested for immunoblotting to detect STAT1-Y701 **(D)** or STAT2 **(E)** levels, respectively. **(F)** The levels of JAK2 in HEK293T cells with expression of ORF3a deletion constructs. Cells were treated similarly to C.

Based on sequence analysis, deletion constructs of ORF3a were prepared to map the domain of ORF3a involved in inducing JAK2 degradation. Four fragments of ORF3a were cloned into the pCAGEN-Flag vector (Figure 7A). Expression of ORF3a truncation constructs in HEK293T cells was confirmed by IFA with Flag antibody (Figure 7B). SOCS1 was detected in HEK293T cells with expression of the ORF3a deletion constructs. The results showed that the cells with ORF3a D1 (aa 1–130), ORF3a D2 (aa 1–200), and ORF3a D4 (aa 70–275) had considerably higher SOCS1 levels than the cells transfected with EV, whereas ORF3a D3 (aa 131–275) had much less effect (Figure 7C). It indicates that the common amino acid domain of all the truncated ORF3a except for D3, aa 70–130, was correlated with the upregulation of SOCS1. The activation and expressions of STAT1 and STAT2 were also determined to evaluate the effect of ORF3a deletion constructs on IFN-activated JAK/STAT signaling. All the ORF3a deletion constructs except D3 resulted in the downregulation of STAT1-Y701 (Figure 7D) and STAT2 (Figure 7E). In addition, the ORF3a D1, D2, and D4 decreased the JAK2 levels (Figure 7F). The results above suggested that aa 70–130 in the middle domain of ORF3a might induce SOCS1 elevation to inhibit JAK/STAT signaling activation and accelerate JAK2 degradation.

## Discussion

Severe acute respiratory syndrome coronavirus 2 has evolved multiple strategies to effectively evade host innate immunity, such as antagonizing IFN production and inhibiting IFN signaling (Hadjadj et al., 2020; Hu et al., 2020; Lei et al., 2020; Xia et al., 2020). In the present study, we discovered that SARS-CoV-2 ORF3a, an accessory protein, upregulated SOCS1 level, which dampens STAT1 activation and mediates JAK2 ubiquitin-proteasomal degradation. Moreover, the middle region of ORF3a (aa 70–130) is associated with the ORF3a-induced SOCS1 elevation.

Previous studies showed that several SARS-CoV-2 proteins are involved in blocking IFN-activated signaling, including viral non-structural proteins (nsp1, 3, 6, 7, 13, and 14), structural proteins (M, N), and accessory proteins (ORF3a, 6, 7, and 8) (Lei et al., 2020; Xia et al., 2020; Hayn et al., 2021; Kumar et al., 2021). However, there are contradictory reports about the effect of ORF3a on IFNα/β-induced ISRE-promoter activation. Xia et al. (2020) reported that SARS-CoV-2 ORF3a significantly suppressed the activation of ISRE, while Lei et al. (2020) and Kumar et al. (2021) showed that ORF3a had minimal effect on ISRE reporters. Therefore, in this study, to evaluate the role of ORF3a on IFN-activated signaling, we conducted a luciferase reporter assay to detect ISRE activation, as well as RT-qPCR and immunoblotting to evaluate IFN-responsive genes. The result showed that ORF3a had significant and consistent inhibitory activity on IFN signaling in the three different detection ways.

Severe acute respiratory syndrome coronavirus 2 proteins inhibit IFN response at multiple levels, including hindering IFN’s bind to its receptor, suppressing JAKs or STATs activation, blocking ISGF3 nuclear translocation, and shutting down antiviral proteins synthesis, etc. For example, nsp14 targets the type I IFN receptor for lysosomal degradation (Hayn et al., 2021) and abolishes ISGs induction *via* translational shutdown (Hsu et al., 2021). Nsp1 dampens ISG induction *via* inducing depletion of TYK2 and STAT2 (Kumar et al., 2021). ORF6 suppresses ISGF3 nuclear translocation through interacting with a component of the nuclear pore complex Nup98 and nuclear importin KPNA2, and the C-terminal region of ORF6 is critical for its antagonistic effect (Lei et al., 2020; Miorin et al., 2020; Xia et al., 2020). Xia et al. (2020) report that ORF3a distinctly impairs STAT1 phosphorylation, consistent with our results. But, the underlying mechanism was not elucidated. We discovered that ORF3a impeded STAT1 activation and nuclear translocation *via* increasing SOCS1 level. SOCS1 is a negative feedback regulator of JAK/STAT signaling. It can disrupt the kinase activity of JAKs by directly interacting with the activation loop of JAKs, and then inhibits the JAKs-mediated STAT1 phosphorylation (Liau et al., 2018).

Several viruses are known to induce a robust expression of host SOCS1, which is hijacked by viruses as an intrinsic virulence factor to evade host immune response and promote virus survival (Akhtar and Benveniste, 2011; Johnson et al., 2020). For example, the influenza A virus (IAV) upregulates SOCS1 to inhibit type I IFN antiviral signaling (Pothlichet et al., 2008). Zika virus promotes the expression of SOCS1 to modulate viral replication (Seong et al., 2020). Coronavirus transmissible gastroenteritis virus (TGEV) and porcine epidemic diarrhea virus (PEDV) increase SOCS1 expression to dampen the type I or type III IFN antiviral response and facilitate viral replication (Ma et al., 2018; Wang et al., 2020). SOCS1 contains a kinase inhibitory region (KIR), which is crucial for its viral virulence effect. Specifically, SOCS1 binds to the kinase activation loop of JAKs through the KIR domain, which results in blockage of STATs activation, consequently inhibits IFN-activated signaling transduction (Liau et al., 2018; Johnson et al., 2020). This study found that SARS-CoV-2 ORF3a increased SOCS1 levels in a dose- and time-dependent manner. Silencing of SOCS1 eliminated the inhibitory effect of ORF3a on IFN-induced STAT1 phosphorylation. It demonstrates that ORF3a upregulating SOCS1 may be one of the reasons for SARS-CoV-2 to antagonize the IFN response. Virus upregulate SOCS1 levels through distinct mechanisms. For example, TGEV, PEDV, respiratory syncytial virus (RSV), and rhinoviruses increase SOCS1 expression by manipulating microRNA levels (such as miR-30a-5p, miR-155, and miR-122) (Zheng et al., 2015; Ma et al., 2018; Wang et al., 2020; Collison et al., 2021). Murine gammaherpesvirus-68 (MHV-68), porcine reproductive and respiratory syndrome virus (PRRSV), and IAV elevate SOCS1 expression *via* different signaling pathways (such as NF-κB signaling, p38/AP-1, and JNK/AP-1 signaling, RIG-I/MAVS/IFNAR1 axis) (Pothlichet et al., 2008; Shen et al., 2018; Luo et al., 2020). The underlying mechanism of SARS-CoV-2 ORF3a upregulates SOCS1 remains unclear. It needs to be elucidated in future studies.

It is reported that a small peptide antagonist of SOCS1/3, pJAK2 (1001–1013), can efficiently block SOCS1/3 inhibitory activity and prevent virus pathogenesis (Johnson et al., 2020). The antagonist corresponds to the activation loop of JAK2 and blocks SOCS function by binding to the KIR region of SOCS1 and SOCS3. There are several preclinical pieces of evidence of the efficacy of the SOCS antagonist as an antiviral. Treatment of keratinocytes with pJAK2 (1001–1013) rendered an antiviral state against herpes simplex virus 1 (Frey et al., 2009). pJAK2 (1001–1013) also inhibited the replication of vaccinia virus (VV) and encephalomyocarditis virus (EMCV) in cell culture, as well as protected mice against lethal VV and EMCV infection (Ahmed et al., 2010). Besides, pJAK2 (1001–1013) was protective in IAV infected cells and influenza virus PR8 lethally infected C57BL/6 mice (Ahmed et al., 2015). In this study, we found that SARS-CoV-2 ORF3a upregulated SOCS1 expression to antagonize IFN signaling. It suggests that SARS-CoV-2 could use SOCS1 as a virulence factor to evade host immune response. Therefore, using SOCS1/3 antagonist to impair SOCS1 inhibitory activity might be an effective preventative/therapeutic against SARS-CoV-2.

Besides direct interaction with the activation loop of JAKs to inhibit signal transduction (Liau et al., 2018), SOCS1 also promotes ubiquitin-proteasomal degradation of target proteins by the SOCS-box at its C-terminal (Linossi and Nicholson, 2012, 2015). Kamizono et al. (2001) pointed out that SOCS1 promotes ubiquitin-dependent degradation of the leukemia-associated TEL-JAK2 fusion protein. In anemia of inflammation (AI), AMPK activation induces SOCS1-mediated JAK2 degradation to relieve AI (Wang et al., 2017). However, there are few reports about SOCS1 mediating protein ubiquitination during virus infection. Du et al. (2020) found that IAV infection induces SOCS1 expression to promote JAK1 ubiquitination, which in turn inhibits host innate immune responses. The present study found that SARS-CoV-2 ORF3a led to JAK2 reduction without influencing its transcript levels. In consideration of ORF3a inducing SOCS1 elevation, we speculated that ORF3a could increase SOCS1 expression to accelerate JAK2 ubiquitin-proteasomal degradation. Indeed, the result showed higher levels of JAK2 ubiquitination in ORF3a expressed cells than in control cells, and silencing of SOCS1 considerably reverses the effect.

ORF3a, a key accessory protein of SARS-CoV-2, is implicated in virulence, infectivity, ion channel formation, and virus release (Issa et al., 2020). It has been reported that ORF3a is involved in autophagy inhibition (Miao et al., 2021; Zhang et al., 2021), inflammasome activation (Xu et al., 2020), nuclear factor-κB signaling suppression (Rui et al., 2021), and apoptosis (Ren et al., 2020). ORF3a possesses an N-terminal, a transmembrane (TM), and a C-terminal domain. Zhang et al. (2021) discovered that the TM domain and C-terminus of ORF3a are necessary for blocking autophagy. Ren et al. (2020) showed that the membrane association is also required for the pro-apoptotic activity of ORF3a. We found that the middle region of ORF3a (aa 70–130) is associated with SOCS1 upregulation. The region is located in the TM domain of ORF3a. It suggests that the TM domain of ORF3a is related to the virus evasion of IFN signaling.

In conclusion, we discovered that SARS-CoV-2 ORF3a inhibits IFN-activated JAK/STAT signaling *via* elevating SOCS1, which hinders the activation of STAT1 and induces the ubiquitin-proteasomal degradation of JAK2. Moreover, the middle domain of ORF3a is responsible for the SOCS1 elevation. This finding provides further insight into SARS-CoV-2 interference with the IFN-mediated antiviral response.

## Data Availability Statement

The original contributions presented in the study are included in the article/supplementary material, further inquiries can be directed to the corresponding authors.

## Author Contributions

RW and EL contributed to the study design. RW was involved in data acquisition and analysis and drafted the manuscript. RW, XY, MC, ZX, WW, and LB performed the experiments. RW, SZ, and EL were involved in the revision of the manuscript. All authors have read and agreed to the published version of the manuscript.

## Conflict of Interest

The authors declare that the research was conducted in the absence of any commercial or financial relationships that could be construed as a potential conflict of interest.

## Publisher’s Note

All claims expressed in this article are solely those of the authors and do not necessarily represent those of their affiliated organizations, or those of the publisher, the editors and the reviewers. Any product that may be evaluated in this article, or claim that may be made by its manufacturer, is not guaranteed or endorsed by the publisher.
